# Antimicrobial activity of red wine and oenological extracts against periodontal pathogens in a validated oral biofilm model

**DOI:** 10.1186/s12906-019-2533-5

**Published:** 2019-06-21

**Authors:** María C. Sánchez, Honorato Ribeiro-Vidal, Adelaida Esteban-Fernández, Begoña Bartolomé, Elena Figuero, María V. Moreno-Arribas, Mariano Sanz, David Herrera

**Affiliations:** 1 ETEP (Etiology and Therapy of Periodontal Diseases) Research Group, University Complutense, Madrid, Spain; 2 Instituto de Investigación en Ciencias de la Alimentación (CIAL), CSIC-UAM, Madrid, Spain; 3 Department of Dental Clinical Specialities (DDCS), Faculty of Odontology, Plaza Ramón y Cajal s/n Ciudad Universitaria, 28040 Madrid, Spain

**Keywords:** Red wine, Oenological extracts, Polyphenols, Periodontal diseases, *P. gingivalis*, *A. actinomycetemcomitans*, *F. nucleatum*

## Abstract

**Background:**

Previous research findings support an antimicrobial effect of polyphenols against a variety of pathogens, but there is no evidence of this effect against periodontal pathogens in complex biofilms. The purpose of this study was to evaluate the antimicrobial activity of red wine and oenological extracts, rich in polyphenols, against the periodontal pathogens *Porphyromonas gingivalis, Aggregatibacter actinomycetemcomitans* and *Fusobacterium nucleatum* and total bacteria growing in an in vitro oral biofilm static model.

**Methods:**

A previously validated biofilm model, including *Streptococcus oralis, Actinomyces naeslundii*, *Veillonella parvula*, *F. nucleatum*, *P. gingivalis* and *A. actinomycetemcomitans* was developed on sterile hydroxyapatite discs. Red wine (and dealcoholized wine), and two polyphenols-rich extracts (from wine and grape seeds) were applied to 72 h biofilms by dipping the discs during 1 and 5 min in the wine solutions and during 30 s and 1 min in the oenological extracts. Resulting biofilms were analyzed by confocal laser scanning microscopy and viable bacteria (colony forming units/mL) were measured by quantitative polymerase chain reaction combined with propidium monoazide. A generalized linear model was constructed to determine the effect of the tested products on the viable bacterial counts of *A. actinomycetemcomitans*, *P. gingivalis* and *F. nucleatum*, as well on the total number of viable bacteria.

**Results:**

The results showed that red wine and dealcoholized red wine caused reduction in viability of total bacteria within the biofilm, with statistically significant reductions in the number of viable *P. gingivalis* after 1 min (*p* = 0.008) and in *A. actinomycetemcomitans* after 5 min of exposure (*p* = 0.011) with red wine. No evidence of relevant antibacterial effect was observed with the oenological extracts, with statistically significant reductions of *F. nucleatum* after 30 s of exposure to both oenological extracts (*p* = 0.001).

**Conclusions:**

Although moderate, the antimicrobial impact observed in the total bacterial counts and counts of *A. actinomycetemcomitans, P. gingivalis* and *F. nucleatum,* encourage further investigations on the potential use of these natural products in the prevention and treatment of periodontal diseases.

## Background

Dental biofilms located at the interface between the teeth and the gingiva are mainly composed of microbial communities encompassing hundreds of different bacterial species. In gingival health, these biofilms are typically comprised of Gram-positive facultative aerobic bacteria, while in presence of gingival inflammation, such as in gingivitis and periodontitis, these biofilms increase in volume and complexity [1]. These changes result in an increment of Gram-negative bacteria and well-recognized pathogens such as *Porphyromonas gingivalis*, *Prevotella intermedia*, *Tannerella forsythia* or *Treponema denticola*, as well as *Aggregatibacter actinomycetemcomitans* [2, 3]. In fact, the etiology of periodontal diseases is currently conceived as a dysbiosis between the bacteria present in dental biofilms and the host response against this bacterial challenge, which would be responsible of the clinical expression of either gingivitis or periodontitis [4].

Prevention and treatment of periodontal diseases mainly consist on strategies to eliminate or reduce these biofilms, either mechanically or chemically (antiseptic and/or systemic or locally applied antimicrobial agents) [5, 6]. However, the widespread use of antibiotics has several unwanted effects, such as the development of bacterial resistances, alterations of the gut microbiota or even direct renal and hepatic damage [7, 8]. Similarly, certain commonly used antiseptics can cause irritation of the oral mucosa, tooth staining or increased dental calculus formation [9]. All these facts indicate the need to develop novel antimicrobial strategies useful for the management of periodontal diseases.

In recent years, scientific evidence has emerged on the potential use of naturally derived phenolic compounds in the prevention/treatment of many chronic diseases, such as cardiovascular, metabolic, or neurodegenerative diseases and, to a lesser extent some cancers [10–13]. Most of these diseases have an inflammatory base and some may be triggered by bacteria. Consequently, there is potential for the use of natural polyphenols, that may exhibit both anti-bacterial and anti-inflammatory properties. It can be therefore hypothesized that phenolic compounds, such as polyphenols could be potentially effective in the prevention and treatment of oral diseases [14].

The anti-inflammatory properties of natural polyphenols have been extensively studied [10], even in relation to the periodontal diseases [14]. In regards to their possible anti-antibacterial effect, previous studies have described different ways of actions; either associated with the ability of polyphenols to generate hydroxyl radicals which would produce H_2_O_2_ and subsequent damage in the bacterial DNA and in its membrane integrity [15], or by altering the structure of the bacterial cell membrane leading to exit of intracellular components, or by changing the intracellular exchange of protons and potassium and phosphate ions [16–18].

Polyphenols are naturally occurring compounds largely found in fruits (i.e., grape, apple, pear or cherry), in cereals, dry fruits, chocolate, and also in beverages (i.e., wine, coffee, beer and tea) [19]. Red wine and grapes are rich sources of phenolic compounds [20]. Its antibacterial action has been evaluated with evidence of inhibitory action on the growth of different *Streptococcus* spp. strains and other bacteria associated with dental caries [21–25]. Also, the effect of polyphenol-rich foods (including several types of tea and wine), lead up as mouth rinses, has been investigated by assessing their inhibitory activity on oral pathogens and on the bacterial adherence to oral tissues [26–28]. However, there are few studies assessing the possible effect of phenolic natural extracts on multi-species biofilms, or specifically on the periodontal pathogens associated with the etiology of periodontal diseases [22, 23, 28–32].

Therefore, the present work aims to evaluate the antimicrobial potential of red wine and dealcoholized red wine, as well as of two oenological extracts (a red wine extract and a grape seed extract), on an in vitro multispecies biofilm model that emulates subgingival biofilms and includes periodontal pathogens such as *P. gingivalis, A. actinomycetemcomitans* and *F. nucleatum*.

## Methods

### Red wines

A young red wine was used in this investigation (var. Pinot Noir, vintage 2010), provided by Bodegas Miguel Torres S.A. (Vilafranca del Penedès, Barcelona, Spain). The phenolic content present in the wine include: total anthocyanins = 0.447 mg of malvidin-3-glucoside mL^− 1^, total catechins = 1.612 mg of (+)-catechin mL^− 1^ and total polyphenols = 1.758 mg of gallic acid equiv. mL^− 1^. The principal individual phenolic compounds found in this wine were flavan-3-ols, flavonols, alcohols, anthocyanins, stilbenes and hydroxycinnamic acids, determined by Ultra-High-Performance Liquid Chromatography-ElectroSpray Ionization-tandem Mass spectrometry (UHPLC-ESI-MS/MS) for other studies [33].

A rotary evaporator was used for the preparation of dealcoholized red wine, removing the EtOH and adding distilled water to reconstitute it until the original volume.

### Oenological extracts

Two commercially available oenological phenolic extracts were used: Provinols™, a red wine extract, kindly supplied by Safic-Alcan Especialidades S.A.U. (Barcelona, Spain) and a grape seed extract, Vitaflavan^®^, kindly provided by Piriou (Les Derives Resiniques & Terpeniques S.A., France). The total phenolic content of the extracts was 474 mg of gallic acid equiv. g^− 1^ for Provinols™ and 629 mg of gallic acid equiv. g^− 1^ for Vitaflavan^®^. The phenolic compositions of both oenological extracts has been determined by UHPLC-ESI-MS/MS in previous studies [34, 35]. Both the wine extract and grape seed extracts were dissolved in distilled water containing 4% dimethyl sulfoxide (DMSO) (v/v), until reaching a final concentration of 20 mg mL^− 1^.

### Bacterial strains and culture conditions

Six bacterial strains, including *Streptococcus oralis* CECT 907 T, *Veillonella parvula* NCTC 11810, *Actinomyces naeslundii* ATCC 19039, *F. nucleatum* DMSZ 20482, *A. actinomycetemcomitans* DSMZ 8324 and *P. gingivalis* ATCC 33277 were used. Bacteria were cultured in blood agar plates (Blood Agar Oxoid No 2; Oxoid, Basingstoke, UK), supplemented with 5% (v/v) sterile horse blood (Oxoid), 5.0 mg L^− 1^ hemin (Sigma, St. Louis, MO, USA) and 1.0 mg L^− 1^ menadione (Merck, Darmstadt, Germany) at 37 °C for 24–72 h in anaerobic conditions (10% H_2_, 10% CO_2_, and balance N_2_).

### Biofilm development

A multi-species in vitro biofilm model was developed as previously described by Sánchez and colleagues [36]. For the inoculum preparation, the microorganisms were individually cultivated in anaerobic conditions on a protein rich medium containing brain-heart infusion (BHI) (Becton, Dickinson and Company, USA) supplemented with 2.5 g L^− 1^ mucin (Oxoid, Thermo Scientific, Hampshire, UK), 1.0 g L^− 1^ yeast extract (Oxoid, Thermo Scientific, Hampshire, UK), 0.1 g L^− 1^ cysteine (Sigma-Aldrich, Barcelona, Spain), 2.0 g L^− 1^ sodium bicarbonate (Merck, NJ, USA), 5.0 mg L^− 1^ hemin (Sigma-Aldrich, Barcelona, Spain), 1.0 mg L^− 1^ menadione (Merck, NJ, USA) and 0.25% (v/v) glutamic acid (Sigma-Aldrich, Barcelona, Spain). The bacterial cultures were harvested at mid-exponential phase (measured by spectrophotometry), and a mixed bacteria suspension in modified BHI medium containing 10^3^ colony-forming units (CFU) mL^− 1^ for *S. oralis*, 10^5^ CFU mL^− 1^ for *V. parvula* and *A. naeslundii*, and 10^6^ CFU mL^− 1^ for *F. nucleatum, A. actinomycetemcomitans* and *P. gingivalis* was prepared. The biofilms were grown on sterile calcium hydroxyapatite (HA) discs of 7 mm of diameter and 1.8 mm (standard deviation, SD = 0.2) of thickness (Clarkson Chromatography Products, Williamsport, PA, USA) discs deposited in 24-wells cell culture plates (Greiner Bio-one, Frickenhausen, Germany), inoculating each well with 1.5 mL of mixed bacteria, for 72 h at 37 °C in anaerobic condition. All assays were performed independently at least three times and in triplicate (*n* = 9).

### Antimicrobial activity

The antimicrobial activity of wines and oenological extracts was examined on 72 h biofilms by determining the reduction in the number of viable CFU mL^− 1^ using the quantitative polymerase chain reaction (qPCR). For the oenological extracts, 30 and 60 s were selected as exposure times since they are bioactive products, commercially available, and for them, the standard exposure times established for other antimicrobial commercially available products (e.g. products with chlorhexidine), was selected [37–39]. On the other hand, in the case of wine solutions, the product was considered as a new possible bioactive agent, evaluated for the first time, therefore, not only the standard 60 s interval was selected as exposure time, but also an “extreme” exposure time of 5 min, with the aim of detecting any possible effect of red wine solutions (dealcoholized or not). Two different protocols were performed:

- For red wine (dealcoholized or not), biofilms were dipped during 1 and 5 min in the wine solutions at room temperature. Phosphate buffer saline (PBS) was used as negative control and, in order to discard a bactericidal effect of the EtOH contained in the wine, also 12% ethanol was applied.
- For the oenological extracts, biofilms were dipped during 30 s and 1 min at room temperature, due to their high phenolic content. PBS was used as negative control, and in order to discard a bactericidal effect of the DMSO used for dissolve the extracts, 4% DMSO solution was also tested.

### Microbiological outcomes

After the antimicrobial treatment, biofilms were sequentially rinsed in 2 mL of sterile PBS three times (immersion time per rinse, 10 s), in order to remove possible remains of the oenological solutions or extracts and unbound bacteria. Then, biofilms were disrupted by vortex for 2 min in 1 mL of PBS. To discriminate between DNA from live and dead bacteria, propidium monoazide (PMA) (Biotium Inc., Hayword, CA, USA) was used. The use of this PMA dye combined with qPCR has shown the ability to detect the DNA from viable bacteria [40]. For this, 100 μM of PMA was added to 250 μL of disaggregated biofilm. Following an incubation period of 10 min at 4 °C in the dark, the samples were subjected to light-exposure for 30 min, using PMA-Lite LED Photolysis Device (Biotium Inc.), and then centrifuged at 12,000 rpm for 3 min prior to DNA extraction.

Bacterial DNA was isolated from all biofilms using a commercial kit ATP Genomic DNA Mini Kit® (ATP biotech. Taipei, Taiwan), following manufacturer’s instructions and the hydrolysis 5’nuclease probe assay qPCR method was used for detecting and quantifying the bacterial DNA. The qPCR amplification was performed following a protocol previously optimized by our research group, using primers and probes targeted against 16S *rRNA* gene [obtained through Life Technologies Invitrogen (Carlsbad, CA, USA)] [41].

Each DNA sample was analysed in duplicate. Quantification cycle (Cq) values, describing the PCR cycle number at which fluorescence rises above the baseline, were determined using the provided software package (LC 480 Software 1.5; Roche Diagnostic GmbH; Mannheim, Germany). Quantification of viable cells by qPCR was based on standard curves. The correlation between Cq values and CFU mL^− 1^ was automatically generated through informatics analysis (LC 480 Software 1.5; Roche).

All assays were developed with a linear quantitative detection range established by the slope range of 3.3–3.5 cycles/log decade, r^2^ > 0.998 and an efficiency range of 1.9–2.0.

### Confocal laser scanning microscopy (CLSM)

Non-invasive confocal imaging of fully hydrated biofilms was carried out using a fixed-stage Ix83 Olympus inverted microscope coupled to an Olympus FV1200 confocal system (Olympus; Shinjuku, Tokyo, Japan). LIVE/DEAD^®^ BacLight™ Bacterial Viability Kit solution (Molecular Probes B. V., Leiden, The Netherlands) was used to stained the biofilms at room temperature. The fluorochromes were incubated (ratio 1:1) during 9 ± 1 min to obtain the optimum fluorescence signal at the corresponding wave lengths (Syto9: 515–530 nm; Propidium Iodide (PI): > 600 nm. The CLSM software was set to take a z-series of scans (xyz) of 1 μm thickness (8 bits, 1024 × 1024 pixels). Image stacks were analyzed by using the Olympus^®^ software (Olympus). Image analysis and live/dead cell ratio (i.e. the area occupied by living cells divided by the area occupied by dead cells) was performed with Fiji software (ImageJ Version 2.0.0-rc-65 / 1.52b, Open source image processing software).

### Statistical analyses

The selected outcome variables to study the antibacterial effect of wine solutions and oenological extracts were the counts of viable bacteria present on the biofilms, expressed as viable CFU mL^− 1^ of *A. actinomycetemcomitans*, *P. gingivalis*, *F. nucleatum* and total bacteria by qPCR, and the live/dead cell ratio of the whole biofilm by CLSM. An experiment-level analysis was performed for each parameter of the study (*n* = 9 for qPCR and *n* = 3 for CLSM results). Shapiro–Wilk goodness-of-fit tests and distribution of data were used to assess normality. Data were expressed as means ± SD.

In the case of the experiments with red wine, the effect of each solution [red wine (dealcoholized or not), PBS and 12% EtOH], the time of exposure (1 or 5 min) and their interaction with the main outcome variable (counts expressed as CFU mL^− 1^ or live/dead cell ratio), was compared by means of a parametric ANOVA test for independent samples, and a general linear model was constructed for each bacterium (*A. actinomycetemcomitans*, *P. gingivalis* and *F. nucleatum*) and for total bacteria for qPCR results and for total bacteria for live/dead cell ratio of whole biofilm obtained by CLSM, using the method of maximum likelihood and Bonferroni corrections for multiple comparisons. A similar model was constructed in the case of the experiments with oenological extracts, in order to compare the effect of each solution (wine extract, grape seed extract, PBS and DMSO), the time of exposure (30 s or 1 min) and their interaction with the main outcome variable (CFU mL^− 1^ and live/dead cell ratio of whole biofilms).

Results were considered statistically significant at *p* < 0.05. A software package (IBM SPSS Statistics 24.0; IBM Corporation, Armonk, NY, USA) was used for all data analysis.

## Results

### Antimicrobial effect of red wine

Table 1 depicts the effects of red wine solutions, dealcoholized or not, compared to PBS and 12% EtOH, on the counts of viable cells of *A. actinomycetemcomitans, P. gingivalis, F. nucleatum* and total bacteria.

**Table 1:** Effect of red wine and dealcoholized red wine on the number of viable bacteria in the in vitro multi-species biofilm [colony forming units, CFU mL^− 1^, obtained by quantitative real-time polymerase chain reaction (qPCR)]. Data are expressed as mean ± standard deviation (SD). PBS: phosphate buffer saline, EtOH: ethanol

	Exposure time (min)	Viable CFU mL^-1^ [mean (SD)] in the biofilm			
		Treatment with PBS	Treatment with the corresponding antimicrobial agent		
			Red wine	Dealcoholized red wine	12% EtOH
*A. actinomycetemcomitans*	1	1.9x10^6^ (7.6x10^5^)	1.8x10^6^ (6.5x10^5^)^**†**^	1.9x10^6^ (1.2x10^6^)^**†**^	8.9x10^5^ (5.6x10^5^)
	5	2.0x10^6^ (2.0x10^6^)	7.7x10^5^ (5.1x10^5^)^*†^	5.1x10^5^ (4.5x10^5^)^*†^	1.1x10^6^ (8.6x10^5^)
*P. gingivalis*	1	1.5x10^5^ (1.3x10^5^)	3.4x10^4^ (3.5x10^4^)^*^	5.9x10^4^ (5.1x10^4^)	1.2x10^5^ (1.4x10^5^)
	5	4.2x10^4^ (3.3x10^4^)	5.2x10^3^ (3.2x10^3^)	5.3x10^3^ (5.6x10^3^)	3.9x10^4^ (1.4x10^4^)
*F. nucleatum*	1	1.4x10^5^ (7.1x10^4^)	1.1x10^5^ (9.1x10^4^)^**†**^	1.2x10^5^ (1.0x10^5^)^**†**^	8.1x10^4^ (8.5x10^4^)
	5	9.2x10^4^ (9.5x10^4^)	3.4x10^4^ (3.1x10^4^)^**†**^	1.9x10^4^ (1.6x10^4^)^**†**^	6.5x10^4^ (5.6x10^4^)
Total bacteria	1	8.2x10^6^ (4.2x10^6^)	3.7x10^6^ (2.7x10^6^)	3.3x10^6^ (2.7x10^6^)	5.9x10^6^ (7.9x10^6^)
	5	7.2x10^6^ (3.1x10^6^)	3.9x10^6^ (7.2x10^6^)	4.0x10^6^ (3.1x10^6^)	3.0x10^6^ (1.9x10^6^)

^*^p < 0.05, significant differences when compared viable CFU mL^-1^ to control biofilms (exposed to PBS)

^†^*p* < 0.05, significant differences when comparing exposure times for an antimicrobial agent

After 1 min of exposure to red wine or dealcoholized red wine, no statistically significant effect was measured on the viable counts of *A. actinomycetemcomitans* (CFU mL^− 1^) (*p* > 0.05) when compared to control biofilms (exposed to PBS). Conversely, after 5 min a significant reduction of viable *A. actinomycetemcomitans* (CFU mL^− 1^) occurred with wine (*p* = 0.053) and dealcoholized red wine (*p* = 0.011) when compared to control biofilms. No statistically significant differences were observed between the two wine solutions at any time (*p* > 0.05). The effect of exposure time (between 1 and 5 min) was however, statistically significant for both red wine (*p* = 0.030), and dealcoholized red wine (*p* = 0.006).

After 1 min exposure to red wine solutions, there were statistically significant reductions in the viable counts of *P. gingivalis* (CFU mL^− 1^) (*p* = 0.008). Measurable reductions also occurred after 5 min of exposure with both red wine and dealcoholized red wine, although no significance differences were observed when compared to biofilms exposed to PBS (*p* > 0.05 in all cases). No statistically significant differences were observed in the effectiveness comparing the two wine solutions at applied times or when comparing exposure times (*p* > 0.05 for all cases).

For *F. nucleatum*, reductions in viable counts were not statistically significant after both 1 and 5 min of exposure (Table 1). No statistically significant differences were observed between the two wine solutions at any time (*p* > 0.05). The effect of exposure time (between 1 and 5 min) was however, statistically significant for both red wine (*p* = 0.035), and dealcoholized red wine (*p* = 0.004).

In regards to biofilm total bacteria, reductions in viable counts were measured (Table 1) after 1 and 5 min of exposure with both solutions, red wine (45.1 and 54.2%, respectively, of viable bacteria after the exposure when compared to control biofilms) and dealcoholized red wine (40.2 and 55.5%, respectively), but differences were not statistically significant (Table 1). No statistically significant differences were observed in the effectiveness when comparing red wine and dealcoholized red wine at 1 or 5 min or when comparing the exposure times (*p* > 0.05 for all cases).

Due to the possible antibacterial activity of EtOH present in the red wine, its effect over the three pathogens and total bacteria was evaluated. Although the treatment with 12% EtOH, emulating the alcoholic content of the wines, resulted in a decrease in total counts (Table 1), no statistically significant differences were observed when compared with PBS (*p* > 0.05 in all cases). No exposure time effect was observed for red wine or dealcoholized red wine, except for *P. gingivalis*, for which the effect of time of exposure (1 min versus 5 min) was statistically significant (*p* = 0.027).

**Fig. 1:**
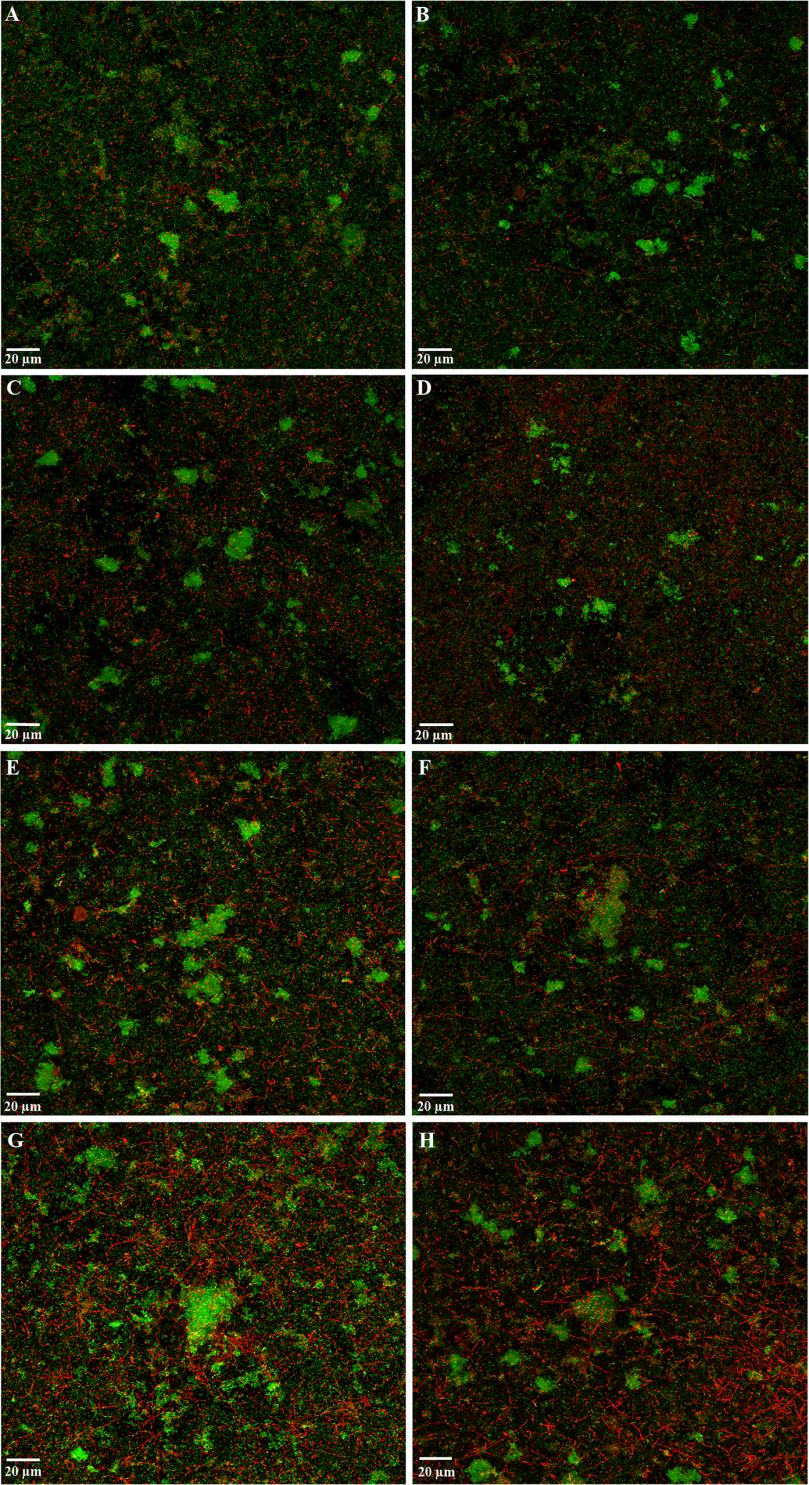
Maximum projection of Confocal Laser Scanning Microscopy (CLSM) images of 72 h biofilms, growth over hydroxyapatite surfaces, stained with LIVE/DEAD^®^ BacLight™ Bacterial Viability Kit, after exposure to: (**a, b**) negative control 1 and 5 min, respectively (phosphate buffer saline, PBS); (**c, d**) 12% ethanol solution 1 and 5 min, respectively; (**e, f**) red wine 1 and 5 min, respectively, and (**g, h**) dealcoholized red wine 1 and 5 min, respectively. Scale bar = 20 μm

After 72 h of incubation, CLSM observation revealed the control HA discs were covered by a mature biofilm, with multicellular aggregates well spread through the surface, showing a structural organization based bacterial communities forming microcolonies, with a live/dead cell ratio of 2.04 ± 0.43 when dipped in PBS for 1 min and 1.10 ± 0.42 for 5 min (Fig. 1 a, b). When biofilms were dipped in red wine for 1 min, a significant decrease in cell viability of the whole biofilm could be observed (*p* < 0.001; Fig. 1 e; Table 2), demonstrating a 0.74 ± 0.05 of live/dead cell ratio, which continued to decrease to 0.53 ± 0.12 after 5 min (Fig. 1 f; Table 2). A significant effect was also observed when exposed to dealcoholized red wine for 1 min (0.84 ± 0.23 of live/dead cell ratio; *p* < 0.001; Table 2) and 5 min (0.52 ± 0.03; *p* > 0.05) (Fig. 1 g, h; Table 2). Visual changes were also appreciated when applying the 12% EtOH solution for 1 and 5 min (live/dead cell ratio of 1.31 ± 0.26 and 0.93 ± 0.12, respectively; *p* = 0.018 after 1 min of exposure) (Fig. 1 c, d; Table 2). No statistically significant differences were observed when comparing red wine and dealcoholized red wine for 1 or 5 min or when comparing exposure times (*p* > 0.05 for all cases).

**Table 2:** Effect of red wine and dealcoholized red wine on the live/dead cell ratio (i.e. the area occupied by living cells divided by the area occupied by dead cells) of the whole biofilm obtained by Confocal Laser Scanning Microscopy (CLSM). PBS: phosphate buffer saline, EtOH: ethanol

Exposure time	Chemical treatment		Mean Difference (I-J)	Std. Error	Sig.^b^	95% Confidence Interval for Difference^b^	
						Lower Bound	Upper Bound
1 min	PBS	12% EtOH	0.727	0.208	0.018^a^	0.101	1.352
		Red wine	1.300	0.208	0.000^a^	0.674	1.926
		Dealcoholized wine	1.197	0.208	0.000^a^	0.571	1.822
	12% EtOH	PBS	-0.727	0.208	0.018^a^	-1.352	-0.101
		Red wine	0.573	0.208	0.084	-0.052	1.199
		Dealcoholized wine	0.470	0.208	0.229	-0.156	1.096
	Red wine	PBS	-1.300	0.208	0.000^a^	-1.926	-0.674
		12% EtOH	-0.573	0.208	0.084	-1.199	0.052
		Dealcoholized wine	-0.103	0.208	1.000	-0.729	0.522
	Dealcoholized wine	PBS	-1.197	0.208	0.000^a^	-1.822	-0.571
		12% EtOH	-0.470	0.208	0.229	-1.096	0.156
		Red wine	0.103	0.208	1.000	-0.522	0.729
5 min	PBS	12% EtOH	0.177	0.208	1.000	-0.449	0.802
		Red wine	0.577	0.208	0.082	-0.049	1.202
		Dealcoholized wine	0.593	0.208	0.069	-0.032	1.219
	12% EtOH	PBS	-0.177	0.208	1.000	-0.802	0.449
		Red wine	0.400	0.208	0.435	-0.226	1.026
		Dealcoholized wine	0.417	0.208	0.374	-0.209	1.042
	Red wine	PBS	-0.577	0.208	0.082	-1.202	0.049
		12% EtOH	-0.400	0.208	0.435	-1.026	0.226
		dealcoholized wine	0.017	0.208	1.000	-0.609	0.642
	Dealcoholized wine	PBS	-0.593	0.208	0.069	-1.219	0.032
		12% EtOH	-0.417	0.208	0.374	-1.042	0.209
		Red wine	-0.017	0.208	1.000	-0.642	0.609

Based on estimated marginal means

^a^The mean difference is significant at the 0.05 level

^b^Adjustment for multiple comparisons: Bonferroni

### Antimicrobial effects of oenological extracts

Table 3 depicts the effects of the two polyphenol-rich extracts, compared to the negative control (PBS) and 4% DMSO, on the number of viable cells of *A. actinomycetemcomitans, P. gingivalis, F. nucleatum* and total bacteria.

**Table 3:** Effect of the red wine phenolic extract (Provinols^TM^), rich in anthocyanins, and the oenological extract from grape seeds (Vitaflavan^®^) on the number of viable bacteria in the *in vitro* multi-species biofilm [colony forming units, CFU mL^-1^, obtained by quantitative real-time polymerase chain reaction (qPCR)]. Data are expressed as means ± standard deviation (SD). PBS: phosphate buffer saline; DMSO: dimethyl sulfoxide

	Exposure time	Viable CFU mL^-1^ [mean (SD)] in the biofilm			
		Treatment with PBS	Treatment with the corresponding antimicrobial agent		
			Wine extract	Grape seeds extract	4% DMSO
*A. actinomycetemcomitans*	30 s	7.2x10^6^ (6.4x10^6^)	5.8x10^6^ (3.8x10^6^)	5.2x10^6^ (5.2x10^6^)	5.6x10^6^ (3.0x10^6^)
	1 min	5.2x10^6^ (3.7x10^6^)	5.0x10^6^ (5.8x10^6^)	2.4x10^6^ (1.2x10^6^)	5.2x10^6^ (4.9x10^6^)
*P. gingivalis*	30 s	1.7x10^6^ (7.0x10^5^)	1.8x10^6^ (1.5x10^6^)^†^	1.3x10^6^ (1.5x10^6^)	1.6x10^6^ (1.8x10^6^)
	1 min	8.9x10^5^ (6.8x10^5^)	5.0x10^5^ (2.6x10^5^)^†^	8.5x10^5^ (4.7x10^5^)	1.0x10^6^ (7.0x10^5^)
*F. nucleatum*	30 s	3.8x10^5^ (3.1x10^5^)	1.0x10^5^ (4.6x10^4^)^*^	1.1x10^5^ (9.2x10^4^)^*^	3.5x10^5^ (1.3x10^5^)^†^
	1 min	1.5x10^5^ (1.0x10^5^)	3.3x10^4^ (2.7x10^4^)	5.4x10^4^ (4.2x10^4^)	1.8x10^5^ (1.5x10^5^)^†^
Total bacteria	30 s	3.6x10^7^ (2.3x10^7^)	2.5x10^7^ (1.5x10^7^)^†^	1.9x10^7^ (1.7x10^7^)	1.9x10^7^ (1.9x10^7^)
	1 min	1.3x10^7^ (1.1x10^7^)	5.5x10^6^ (4.7x10^6^)^†^	5.6x10^6^ (3.4x10^6^)	1.1x10^7^ (6.2x10^6^)

^*^p < 0.05, significant differences when compared viable CFU mL^-1^ to control biofilms (exposed to PBS)

^†^*p* < 0.05, significant differences when comparing exposure times for an antimicrobial agent

After 30 s and 1 min exposure to the wine and grape seed extracts, there was a reduction in the viable counts of *A. actinomycetemcomitans,* although statistically significant differences were not detected (Table 3). Comparisons between both extract solutions or between the times of exposure for each extract were not statistically significant (*p* > 0.05 in all cases).

Similarly, no significant effect on viable counts of *P. gingivalis* was observed after exposure to the wine and grape seed extracts during 30 s (Table 3). The number of viable *P. gingivalis* showed reductions when biofilms were treated for 1 min with the wine extract, but not with the grape seed extract (*p* > 0.05 in both cases). No statistically significant differences were observed between the effect reached by the two oenological extracts at any time (*p* > 0.05 in both cases). The effect of time of exposure (30 s *versus* 1 min) was statistically significant for the wine extract (*p* = 0.014), but not for the grape seed extract (*p* = 0.395).

For *F. nucleatum*, 30 s of exposure to both oenological extracts significantly reduced viable counts (*p* = 0.001, in both cases) (Table 3). However, after 1 min of exposure to both, although the reduction was maintained, no statistically differences were reached; although the oenological extract showed more effect on *F. nucleatum*. Similarly, no significant effect was observed when comparing the effect of both extracts at any time or the time of exposure for each one (*p* > 0.05 in all cases).

Regarding the total counts of bacteria included in the biofilm, 30 s and 1 min of contact with both, the wine and grape seed extracts, caused a slight reduction in the number of viable counts, but differences were not statistically significant. Similarly, no significant differences were observed between the two extracts at any time. The effect of time of exposure (30 s versus 1 min) was statistically significant for the wine extract (*p* = 0.005), but not for the grape seed extract (*p* = 0.057).

Due to the possible antibacterial activity of DMSO, its effect on the tested bacterial species and total bacteria was evaluated. It was observed that the treatment with 4% DMSO (v/v), concentration used to solubilize the extracts, had no effect on the bacterial cell viability (Table 3). There were no statistically significant differences when compared with PBS (*p* > 0.05 in all cases), or between exposure time (*p* > 0.05 in all cases) except for *F. nucleatum*, for which the effect of exposure time (30 s versus 1 min) was statistically significant (*p* = 0.012).

**Fig. 2:**
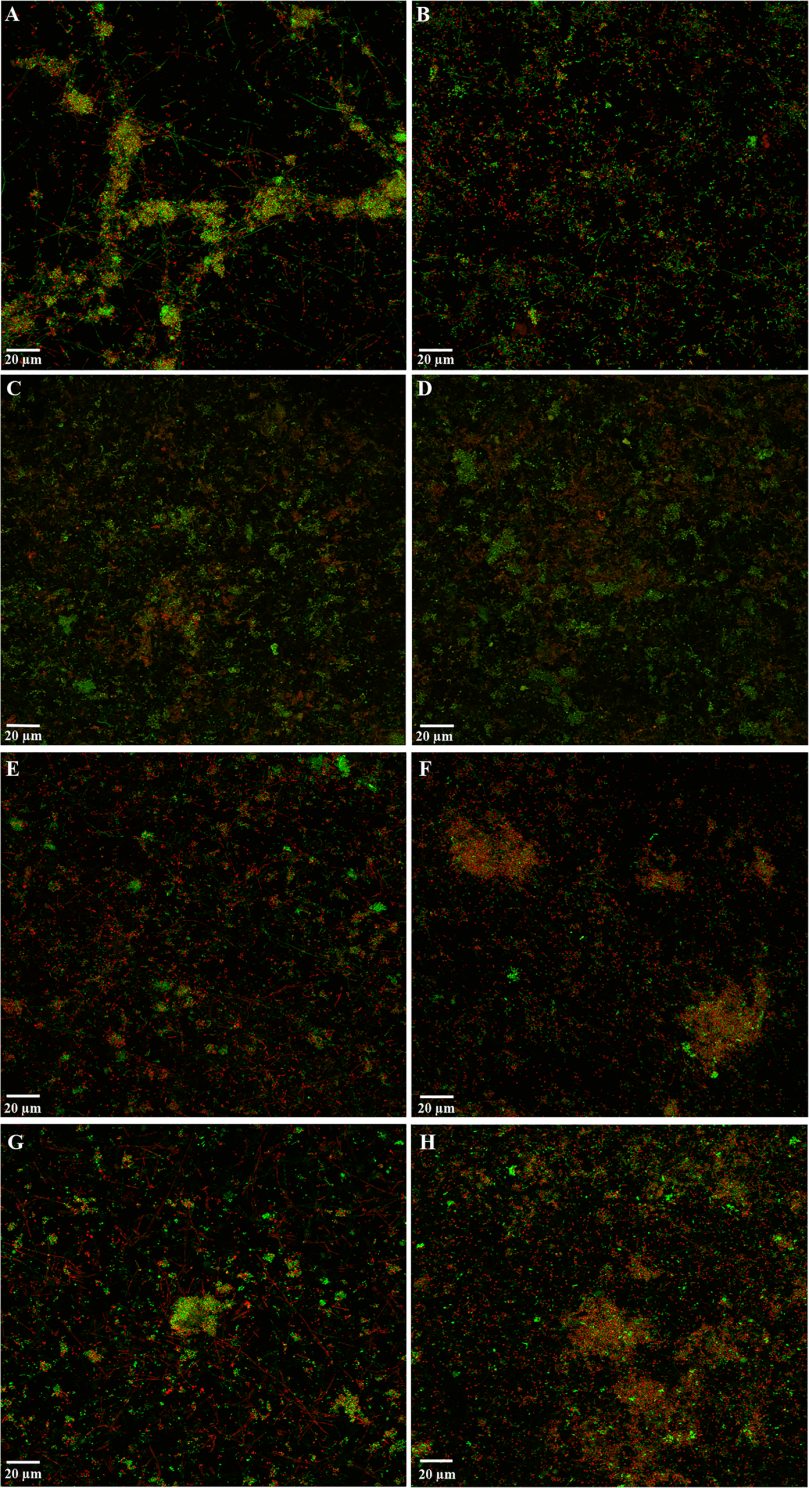
Maximum projection of Confocal Laser Scanning Microscopy (CLSM) images of 72 h biofilms, growth over hydroxyapatite surfaces, stained with LIVE/DEAD^®^ BacLight™ Bacterial Viability Kit, after exposure to: (**a, b**) negative control 30 s and 1 min, respectively (phosphate buffer saline, PBS); (**c, d**) 4% dimethyl sulfoxide (DMSO) solution 30 s and 1 min, respectively; (**e, f**) wine extract 30 s and 1 min, respectively (Provinols™, 20 g L^− 1^); and (**g, h**) grape seed extract (Vitaflavan^®^, 20 g L^− 1^). Scale bar = 20 μm

The CLSM analysis showed that, after 72 h of incubation on HA surfaces, the biofilm covered the disc surface as multicellular aggregates, exhibited a live/dead cell ratio of 1.13 ± 0.50 when dipped for 30 s and 1.10 ± 0.16 for 1 min in PBS (Control biofilms; Fig. 2 a, b). It could be observed that after 30 s exposures to both oenological extracts, cell vitality slightly decreased in the biofilms (live/dead cell ratio of 0.77 ± 0.24 for wine extract and 1.20 ± 0.20 for the grape seed extract; *p* > 0.05 in both cases) (Fig. 2 e, g; Table 4). In the same way, after 1 min exposure to the wine extract (Fig. 2 f) and the grape seed extract (Fig. 2 h), no reduction in viability was measured by CLSM (viability ratio 1.21 ± 0.30 and 1.30 ± 0.47, respectively; *p* > 0.05; Table 4). No visual changes were observed when applying 4% DMSO solution for 30 s and 1 min (viability ratio of 0.87 ± 0.30 and 1.07 ± 0.09, respectively) (Fig. 2 c, d; Table 4). No statistically significant differences were observed when comparing wine and grape seed extracts at 30 s or 1 min or when comparing the exposure times (*p* > 0.05 for all cases).

**Table 4:** Effect of the red wine phenolic extract (Provinols^TM^), rich in anthocyanins, and the oenological extract from grape seeds (Vitaflavan^®^) on the live/dead cell ratio (i.e. the area occupied by living cells divided by the area occupied by dead cells) of the whole biofilm obtained by Confocal Laser Scanning Microscopy (CLSM). PBS: phosphate buffer saline, DMSO: dimethyl sulfoxide

Exposure time	Chemical treatment		Mean Difference (I-J)	Std. Error	Sig.^a^	95% Confidence Interval for Difference^a^	
						Lower Bound	Upper Bound
30 s	PBS	4% DMSO	0.250	0.256	1.000	-0.520	1.020
		Wine extract	0.353	0.256	1.000	-0.416	1.123
		Grape seeds extract	-0.130	0.256	1.000	-0.900	0.640
	4% DMSO	PBS	-0.250	0.256	1.000	-1.020	0.520
		Wine extract	0.103	0.256	1.000	-0.666	0.873
		Grape seeds extract	-0.380	0.256	0.941	-1.150	0.390
	Wine extract	PBS	-0.353	0.256	1.000	-1.123	0.416
		4% DMSO	-0.103	0.256	1.000	-0.873	0.666
		Grape seeds extract	-0.483	0.256	0.463	-1.253	0.286
	Grape seeds extract	PBS	0.130	0.256	1.000	-0.640	0.900
		4% DMSO	0.380	0.256	0.941	-0.390	1.150
		Wine extract	0.483	0.256	0.463	-0.286	1.253
1 min	PBS	4% DMSO	0.030	0.256	1.000	-0.740	0.800
		Wine extract	-0.110	0.256	1.000	-0.880	0.660
		Grape seeds extract	-0.197	0.256	1.000	-0.966	0.573
	4% DMSO	PBS	-0.030	0.256	1.000	-0.800	0.740
		Wine extract	-0.140	0.256	1.000	-0.910	0.630
		Grape seeds extract	-0.227	0.256	1.000	-0.996	0.543
	Wine extract	PBS	0.110	0.256	1.000	-0.660	0.880
		4% DMSO	0.140	0.256	1.000	-0.630	0.910
		Grape seeds extract	-0.087	0.256	1.000	-0.856	0.683
	Grape seeds extract	PBS	0.197	0.256	1.000	-0.573	0.966
		4% DMSO	0.227	0.256	1.000	-0.543	0.996
		Wine extract	0.087	0.256	1.000	-0.683	0.856

Based on estimated marginal means

^a^Adjustment for multiple comparisons: Bonferroni

## Discussion

In the present study, the effect of red wine and oenological extracts in a validated oral biofilm model has been studied, demonstrating that wine solutions (dealcoholized or not) had a greater antimicrobial effects against *A. actinomycetemcomitans* and *P. gingivalis* when compared to the polyphenol rich oenological extracts. When comparing the oenological extracts, wine extract was more active against *P. gingivalis* and *F. nucleatum,* and the grape seed extract against *F. nucleatum.* In regards to the effects on total biofilm bacteria, wine solutions (dealcoholized or not) showed significant reductions in the live/dead cell ratios, in contrast, the oenological extracts did not evidence a relevant antibacterial effect.

Previous in vitro studies evaluating the antimicrobial effect of phenolic compounds from wines and oenological extracts have demonstrated significant effects against selected Gram-positive and Gram-negative pathogenic bacteria [42], enteric pathogens [43], pathogenic bacteria associated with respiratory diseases [44], or gut commensal, probiotic and pathogenic bacteria [45]. In the oral cavity, Toukairin and colleagues [46] reported that polyphenols, extracted from seeds and skin of wine grapes, had antibacterial effects against certain cariogenic bacteria, mainly through inhibition of the adherence of *S. mutans* and other streptococci. Similarly, Cueva and colleagues [44] incubated planktonic pure cultures of *S. mutans* and *S sobrinus* with flavan-3-ols precursors, (+)-catechin and (−)-epicatechin (compounds present in the grape seed extract employed) and reported significant inhibition of bacterial growth. Daglia and colleagues studied the antiseptic effect of dealcoholized red wine in comparison with white wines, demonstrating a stronger action of red wines against oral streptococci, what reinforces the possible role of anthocyanins as bacteriostatic agents [25]. Recently, Esteban-Fernández and colleagues [21] showed antimicrobial activity against *P. gingivalis, F. nucleatum* and *S. mutans* growing planktonically when exposed to two wine phenolic compounds (caffeic and p-coumaric acids) and the same red wine and grape seed extracts (Provinols™ and Vitaflavan^®^, respectively) used in the present study.

These studies, however, have focused the study of their antimicrobial effect on species commonly detected in supragingival plaque, such as *S. mutans, S. sobrinus* or *Lactobacillus* spp*.,* but not against the periodontal pathogens usually present in the subgingival microenvironment. Furthermore, most have used planktonic pure cultures and therefore, the reported effects could not be easy to be transferred to the oral environment, where bacteria live in highly complex communities, forming biofilms [47]. As mentioned above, Esteban-Fernández and colleagues [21], established the minimum inhibitory (MIC) and minimum bactericidal (MBC) concentrations (MIC/MBC) for *P. gingivalis* to Provinols™ and Vitaflavan^®^ of 500/≥1000 μg mL^− 1^ for both extracts, and for *F. nucleatum* of 500/1000 μg mL^− 1^ also for the referred extracts. However, in the present study, the observed antimicrobial activity can be considered as moderate against *P. gingivalis* and only statistically significant for *F. nucleatum* with both extracts, even at a high concentration (20,000 μg mL^− 1^). These findings reinforce the importance of using biofilms models when testing antimicrobial activity, since bacterial cells in biofilms express different phenotypes, with greater resistance to antimicrobial agents [47–50]. Some studies have reported that the MIC of a bacteria can increase between 2 and 1000 times in a biofilm, when compared to the planktonic state [50], while other authors described 250 times greater MIC values for the same species growing in a biofilm when compared to planktonic state [51]. Sedlack and colleagues [51] described that bacterial resistance to antimicrobials appeared to be related to the maturation of the biofilms, since they demonstrated a progressive increase in resistance to the antibiotics as they matured, with a maximum resistance coinciding with the stationary phase of the growth of the biofilm. Therefore, the current work represents a further step in the study of the possible effects of polyphenols from red wine and oenological extracts in the management of periodontal diseases.

The results from the present study agree with those reported by Furiga and colleagues evaluating the activity of various extracts obtained from *Vitis vinifera* (Vitaceae) on a biofilm model composed of *S. mutans, S. sobrinus, Lactobacillus rhamnosus, P. gingivalis*, and *F. nucleatum* [22, 23]; and with those published by Muñoz-Gonzalez and colleagues [28], describing the beneficial the bactericidal activity against *A. oris, F. nucleatum*, or *S. oralis*. of red wine and dealcoholized red wine.

## Conclusions

This investigation has shown that the use of red wine and wine-derived extracts had a moderate antimicrobial impact in the total bacterial counts and counts of *A. actinomycetemcomitans, P. gingivalis* and *F. nucleatum,* when tested in an in vitro multi-species biofilm model. Although the antibacterial effects of red wine and wine-derived extracts was observed, at least 2 to 3-log reduction of bacterial count would be necessary to ascertain the efficacy and/or availability of these tested agents as antibacterial agents. These results encourage further investigations on the potential use of natural agents in the prevention and treatment of periodontal diseases.

## Data Availability

The data sets used and/or analyzed during the current study available from the corresponding author on reasonable request.

## Abbreviations

BHI: Brain heart infusion culture medium
CFU: Colony-forming units
CLSM: Confocal laser scanning microscopy
DMSO: Dimethyl sulfoxide
DNA: Deoxyribonucleic acid
EtOH: Ethanol
HA: Hydroxyapatite
MBC: Minimum bactericidal concentration
MIC: Minimum inhibitory concentration
PBS: Phospate buffer saline
PMA: Propidium monoazide
qPCR: Quantitative polymerase chain reaction
UHPLC-ESI-MS/MS: Ultra-high-performance liquid chromatography-electrospray ionization-tandem mass spectrometry method
